# T-Bet Deficiency Attenuates Bile Duct Injury in Experimental Biliary Atresia

**DOI:** 10.3390/cells10123461

**Published:** 2021-12-08

**Authors:** Sujit K. Mohanty, Bryan Donnelly, Haley Temple, Alexander Bondoc, Monica McNeal, Greg Tiao

**Affiliations:** 1 Endemic Poultry Viral Diseases Research Unit, Southeast Poultry Research Laboratory, United States National Poultry Research Center, USDA/ARS, Athens, GA 30605, USA; Sujit.Mohanty@usda.gov; 2 Department of Pediatric and Thoracic Surgery, Cincinnati Children’s Hospital Medical Center, Cincinnati, OH 45229, USA; Bryan.Donnelly@cchmc.org (B.D.); Haley.Temple@cchmc.org (H.T.); Alex.Bondoc@cchmc.org (A.B.); 3 Division of Infectious Diseases, Cincinnati Children’s Hospital Medical Center, Cincinnati, OH 45229, USA; Monica.McNeal@cchmc.org; 4 Department of Pediatrics, University of Cincinnati College of Medicine, Cincinnati, OH 45267, USA

**Keywords:** cholestasis, bile duct, STAT1, RRV, T-bet

## Abstract

Biliary atresia (BA) is an obstructive neonatal cholangiopathy leading to liver cirrhosis and end stage liver disease. A Kasai portoenterostomy may restore biliary drainage, but most patients ultimately require liver transplantation for survival. At diagnosis, immune cells within the liver of patients with BA demonstrate a T-helper 1 (Th1) inflammatory profile similar to rhesus rotavirus (RRV)-infected mice livers developing BA. The transcription factor Tbx21 (T-bet) is essential for induction of a Th1 immune response in both the adaptive and innate immune system. Here we used animals with targeted deletion of the T-bet gene to determine its role in the progression of BA. Infection of newborn T-bet knockout (KO) pups with RRV resulted in a decreased Th1 inflammatory chemokine/cytokine profile when compared to infected wild-type mice. Analysis of the mononuclear cells profile from T-bet KO mice revealed both a significant decrease in the total number of CD3, CD4, and CD8 T cells and their effector molecules granzyme A, perforin, and FasL. Even though the percentage of T-bet KO mice displaying symptoms of an obstructive cholangiopathy and overall mortality rate was not different compared to wild-type mice, the extrahepatic bile ducts of T-bet KO mice remained patent.

## 1. Introduction

Biliary atresia (BA) is a progressive obliterative cholangiopathy caused by an inflammatory obstruction of the extrahepatic bile duct. Biliary atresia affects between 1 in 5000 to 18,000 [1] infants and is the leading indication for liver transplantation in the pediatric population, accounting for around 50% of all transplants [2,3]. A surgical bypass called a Kasai portoenterostomy may restore biliary drainage in some infants, but most progress to cirrhosis, end-stage liver disease, and ultimately require liver transplantation at some point in their life [4].

The etiology of this disease is still unknown. One proposed mechanism is a perinatal viral infection triggering an unchecked inflammatory response progressing to obliteration of the extrahepatic bile duct. Viruses including rotavirus (RV) group C [5], reovirus type 3 [6], cytomegalovirus [7], Epstein–Barr virus [8], and human papillomavirus [9] have been detected in explanted livers of BA patients providing evidence to support this mechanism. Additionally, a mouse model of BA exists utilizing the infection of newborn BALB/c pups with Rhesus rotavirus (RRV) resulting in obstruction of the extrahepatic biliary tree mirroring the human disease. Injected pups develop symptoms of biliary obstruction including jaundice, bilirubinuria, and acholic stools, with a resulting mortality rate of 90–100% by day of life (DOL) 14 [10]. The histological and morphological changes in the extrahepatic bile duct, along with the temporal susceptibility of this model, parallel those seen in human BA [11,12].

The immune profile of mononuclear cells found within the livers of infants afflicted with biliary atresia express a T helper (Th) 1 inflammatory response which is thought to cause the inflammation of the extrahepatic bile duct that ultimately leads to the disease [13]. This Th1 profile is similar to that expressed in the RRV-induced murine model of biliary atresia that was described previously. This Th1 response produces the cytokine IFN-γ and previously published data show that the absence of IFN-γ improves cholestasis by decreasing lymphocyte tropism for bile ducts, and the absence of CD8 T cells and natural killer (NK) cells prevents duct obstruction [14,15].

The transcription factor Tbx21 (T-bet) is an important element in most cell types of both adaptive and innate immune systems which is required for (a) determining the fate of Th1 lineage commitment, (b) generation of type 1 immunity, (c) IFN-γ production, and (d) establishing the effector function of CD8 T and NK cells. Therefore, we hypothesized that loss of T-bet would suppress the proinflammatory immune response and henceforth prevent extrahepatic bile duct obstruction in murine BA. Loss of Th1 response was obtained by using mice carrying an inactivation of the Tbx21 gene encoding the transcription factor T-bet (T-bet knockout (KO) mice). Although the T-bet KO mice displayed symptoms of an obstructive cholangiopathy, most mice had a patent extrahepatic bile duct.

## 2. Materials and Methods

### 2.1. Viruses, Cells, and Animals

African green monkey kidney cells, MA104 cells, (BioWhittaker, Walkersville, MD, USA) were cultured as described [16]. The simian rotavirus strain, RRV (generously provided by H. Greenberg, Stanford University, Palo Alto, CA, USA), was propagated in MA104 cells as previously described [11,17].

The mice utilized in these experiments were wild-type BALB/c mice (Envigo Labs, Indianapolis, IN, USA) and BALB/c mice with a genetic inactivation of T-bet (Laurie H Glimcher, Harvard University, Cambridge, MA, USA). Mice were housed in micro isolator cages in a virus-free environment with free access to sterilized chow and water. All animal research was performed in accordance with regulations and protocols approved by the Institutional Animal Care and Use Committee at Cincinnati Children’s Hospital Medical Center.

### 2.2. Experimental Model of Biliary Atresia

Mice underwent intraperitoneal (ip) inoculation on DOL 1 with RRV at a dosage of 1.25 × 10^6^ focus-forming unit (FFU) per gram weight. Mice were observed daily for obstructive symptomology including weight loss, acholic stools, jaundice, and bilirubinuria measured through commercially available urine dipsticks (Bayer Co., Elkhart, IN, USA). Their liver and bile ducts were harvested at 3, 5, 7, 8, 10, and 12 days post inoculation for virus titers, flow cytometry, RNA isolations, and histologic analysis.

### 2.3. RNA Isolation and Real-Time PCR for Detection of Chemokines and Cytokines

Pups were injected with RRV or saline on DOL 1. Subsets of mice had their livers harvested 3, 5, 8, and 12 days after injection. RNeasy Mini Kit (Qiagen, Germantown, MD, USA) was utilized to extract total RNA from the tissues according to the manufacturer’s instructions. cDNA pools were generated using standard reagents (Invitrogen, Carlsbad, CA, USA) and the expression of IFN-γ, TNF-α, IL-6, IL-13, IL-17, IL-21, and IL-1β were quantified by real-time PCR on an Mx-3000 Multiplex Quantitative PCR (Stratagene, La Jolla, CA, USA) and normalized to glyceraldehyde-3-phosphate dehydrogenase (GAPDH) as previously described [18].

### 2.4. Flow Cytometry for CD3, CD4, and CD8 T Cells

Immune cell populations were isolated from the livers of 7 days post inoculated pups and analyzed by flow cytometry as previously described [19].

### 2.5. Histologic Processing of Specimens

Livers were harvested at 7 and 10 days post injection (DPI) and bile ducts on 12 DPI of either virus- or saline-injected mice. Tissues were fixed in 10% formalin, paraffin embedded, and sectioned at 5 µm serially as previously described [20]. Standard techniques were used to stain slides with hematoxylin and eosin (H&E).

Fixed liver slides were baked at 65 °C for 30 min, de-paraffined in 100% xylene, and rehydrated through a series of ethanol steps. Antigen retrieval was performed in Target Retrieval Solution, pH 6, (Dako, Santa Clara, CA, USA), and cooked in a pressure cooker for 30 min. Slides were blocked in a 3% hydrogen peroxide solution for 15 min, washed in water, and placed in 1× phosphate buffered saline (PBS). Tissues were outlined with a PAP pen and blocked with 10% normal goat serum or 10% normal rabbit serum in 1× PBS and incubated for 60 min at room temperature followed by a milk block solution for 10 min at room temperature. Next, either rat anti-mouse CD3 antibody (1:100, Bio-Rad, Hercules, CA, USA), anti-Integrin alpha 2 (1:100, Bio-Rad, Hercules, CA, USA), or anti-Ly6G (1:500, Bio-Rad, Hercules, CA, USA) were added in PBS + 1% BSA and incubated at 4 °C overnight. Slides were then washed in 1× PBS followed by application of a biotinylated goat anti-rabbit antibody (Vector Laboratories, Burlingame, CA, USA) or rabbit anti-mouse (Vector Laboratories, Burlingame, CA, USA) diluted in 10 mL of 1× PBS (1:1000 dilution) and incubated overnight at 4 °C. After incubation, slides were washed 4 times with PBS followed by applicate of 100 µL of pre-prepared Avidin-Biotin complex (ABC) solution (Vector Laboratories, Burlingame, CA, USA) to slides and incubated at room temperature for 50 min. Slides were again washed with 1× PBS followed by addition of 100 µL of 3,3′-diaminobenzidine (DAB) solution (Vector Laboratories, Burlingame, CA, USA) (4 mL of stock buffer, 7.5 mL of DAB solution and 6 mL of H_2_O_2_ in 200 mL of water) to the slides and incubated for 30 s at room temperature, then washed immediately with running water followed by 3 washes of PBS. The slides were then counterstained with hematoxylin for 30 s and rinsed with water. The slides were then dehydrated through a series of ethanol steps and, finally, cover slips were applied using Cytoseal (Thermo Scientific, Waltham, MA, USA).

### 2.6. Quantification of Virus Infectivity

Bile ducts harvested 7 and 10 days post inoculation were analyzed for the presence of infectious RRV by fluorescent focus forming assay (FFA) as previously described [18].

### 2.7. Statistical Analysis

Analysis of continuous variables was performed by analysis of variance (ANOVA) with post-hoc testing and expressed as means ± standard errors (SEM) where appropriate. Results for non-continuous variables were analyzed by the chi-square and Fisher exact tests. *p* values < 0.05 were considered significant.

## 3. Results

### 3.1. T-Bet KO and Wild-Type Mice Display Similar Phenotype Following RRV Inoculation

**Figure 1:**
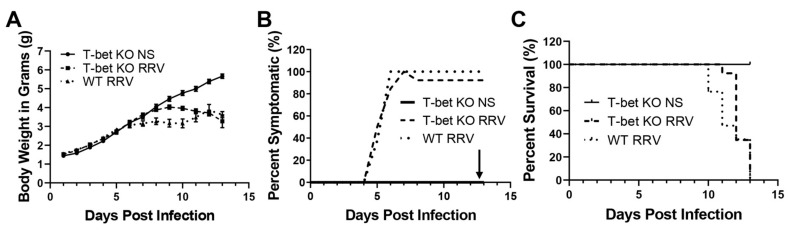
In vivo effect of T-bet KO on the murine model of biliary atresia. RRV infection resulted in a reduced growth curve in both T-bet KO mice and wild-type (WT) mice compared to normal saline (NS) controls (**A**). Similarly, there was no difference in symptoms with T-bet KO mice (90%) developing symptoms compared to WT mice (100%) (**B**). A mortality rate of 100% was witnessed by both WT and T-bet KO mice by DOL 15 (**C**).

Wild-type (WT) (*n* = 17) and T-bet KO BALB/c neonatal mice were inoculated with RRV (1.5 × 10^6^ ffu) (*n* = 26) or normal saline (NS) (*n* = 8) intraperitoneally in the first 24 h after birth. The mice were monitored daily for obstructive symptomatology including weight loss, bilirubinuria, jaundice and acholic stools. The inoculation with RRV resulted in the development of acholic stools, bilirubinuria by 7 days and poor growth by 13 days of age. There was no significant difference in weight loss observed between RRV-infected WT and T-bet KO mice or in symptoms of an obstructive cholangiopathy, with 90% of T-bet KO mice developing symptoms compared to 100% of WT mice (Figure 1A,B). By 15 DPI both the RRV-infected T-bet KO and WT mice had a mortality rate of 100% (Figure 1C).

### 3.2. T-Bet KO Mice Displayed a Decreased Expression of Th1 Proinflammatory Cytokines and Chemokines Following RRV Infection

**Figure 2:**
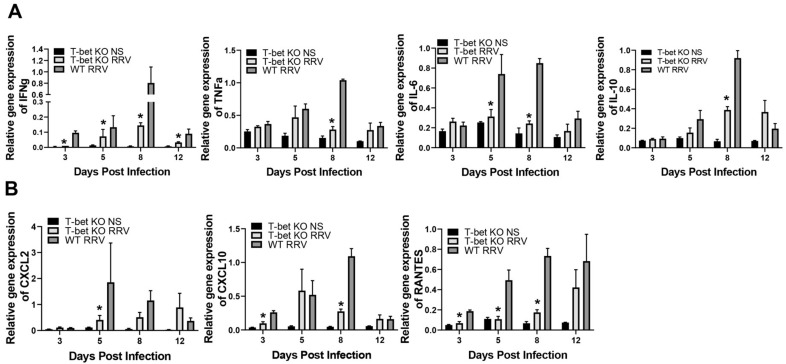
Hepatic expression of Th1 proinflammatory cytokines and chemokines. There was a decrease in hepatic expression of Th1 proinflammatory cytokines IFN-γ, TNF-α, IL-6, and IL-1β (**A**) and chemokines CXCL2, RANTES, and CXCL10 following RRV infection in T-bet KO mice compared to RRV-infected wild-type (WT) mice (**B**); * *p* < 0.05 when compared between RRV-infected T-bet KO mice and WT mice. Injection of normal saline (NS) was used as control.

The presence of Th1 proinflammatory cytokines has previously been demonstrated to trigger inflammation of the bile ducts in the murine model of BA [21,22], therefore we wanted to quantify the expression levels of the proinflammatory cytokines IFN-γ, TNF-α, IL-6, and IL-1β in RRV infection. Following RRV inoculation, livers were harvested at 3, 5, 8, and 12 days. Liver mRNA expression was quantified by real-time PCR. RRV-infected T-bet KO mice expressed statistically lower levels of all four cytokines compared to RRV-infected WT mice (Figure 2A). Similarly, the expression of chemokines CXCL2, CXCL10, and RANTES were also significantly reduced following RRV infection of T-bet KO mice compared to WT mice (Figure 2B).

### 3.3. T-Bet KO Mice Showed Increased Expression of Th2 Anti-Inflammatory Cytokines and Chemokines Following RRV Infection

**Figure 3:**
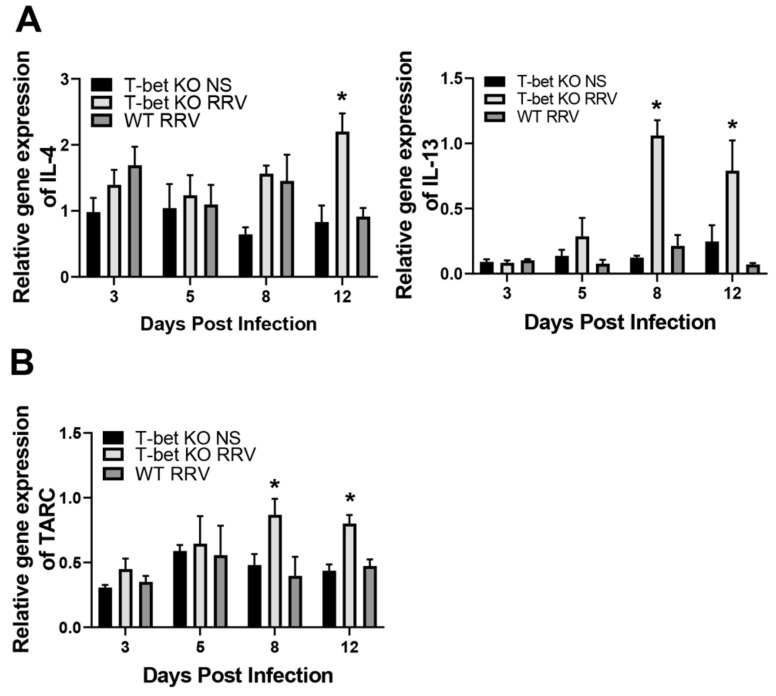
Hepatic expression of Th2 cytokines and chemokines. There was an increase in hepatic expression of Th2 cytokines, IL-4 and IL-13 following RRV infection in T-bet KO mice compared to RRV-infected wild-type (WT) mice (**A**) along with the Th2 chemokine TARC (**B**); * *p* < 0.05 when compared between RRV-infected T-bet KO mice and WT mice. Injection of normal saline (NS) was used as control.

With a decrease in Th1 response, we next wanted to investigate the effect on Th2 response in these T-bet knock-out mice following RRV infection. In contrast to WT infected pups, the RRV-infected T-bet KO mice displayed an increase in both Th2 cytokines IL-4 and IL-13 (Figure 3A) along with the Th2 chemokine TARC (Figure 3B).

### 3.4. Decreased Number of T Lymphocytes in T-Bet KO Livers after RRV Challenge

**Figure 4:**
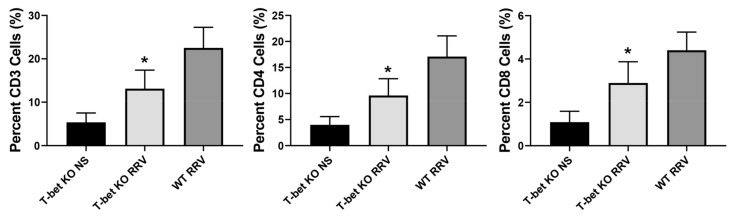
Quantification of liver mononuclear cells (MNC). Flow cytometry of MNC shows decreased number of CD3, CD4, and CD8 T cells in T-bet KO mice compared to RRV-infected wild-type (WT) mice; * *p* < 0.05 when compared between RRV-infected T-bet KO mice and WT mice. Injection of normal saline (NS) was used as control.

To directly examine the role of T-bet in the control of the neonatal Th1 response and to determine if the decrease in Th1 response was due to an overall difference in mononuclear cells (MNC), MNCs were isolated from the livers at 3, 5, 8, and 12 days following RRV infection and flow cytometry was performed. T-bet KO mice displayed a significant decrease in the total number of CD3, CD4, and CD8 T cells when compared to WT mice (Figure 4).

### 3.5. Mononuclear Cells from RRV-Infected T-Bet KO Mice Demonstrate Decreased Expression of Effector Molecules

**Figure 5:**
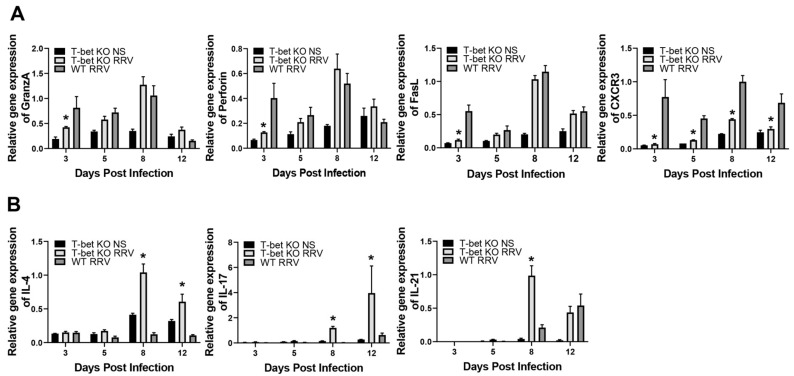
Quantification of effector molecules in liver mononuclear cells (MNC). There was a decreased expression of effector molecules granzyme A, perforin FasL, and CXCR3 in liver MNC early after RRV infection in T-bet KO mice compared to RRV-infected wild-type (WT) mice (**A**). The MNC from T-bet KO mice had increased levels of expression of IL-4, IL-17, and IL-21 when compared to RRV-infected WT mice (**B**). * *p* < 0.05 when compared between RRV-infected T-bet KO mice and WT mice. Injection of normal saline (NS) was used as control.

To further evaluate the inactivation of T-bet on the MNCs in the murine model of BA, we isolated the livers from infected pups and assessed expression levels of effector molecules previously demonstrated to be involved in the induction of the disease [23]. RRV-infected T-bet KO mice exhibited a decreased expression of the effector molecules granzyme A, perforin, and FasL, all significantly lowered at day 3, with CXCR3 significantly reduced at days 3, 5, 8, and 12 when compared to the RRV-infected WT mice (Figure 5A). Similar to the expression witnessed in liver samples, the expression of Th2 and Th17 cytokines, IL-4, IL-17, and IL-21 were also increased in the MNC from T-bet KO mice (Figure 5B).

### 3.6. RRV-Infected T-Bet KO Livers Have a Predominance Infiltration of Neutrophils

**Figure 6:**
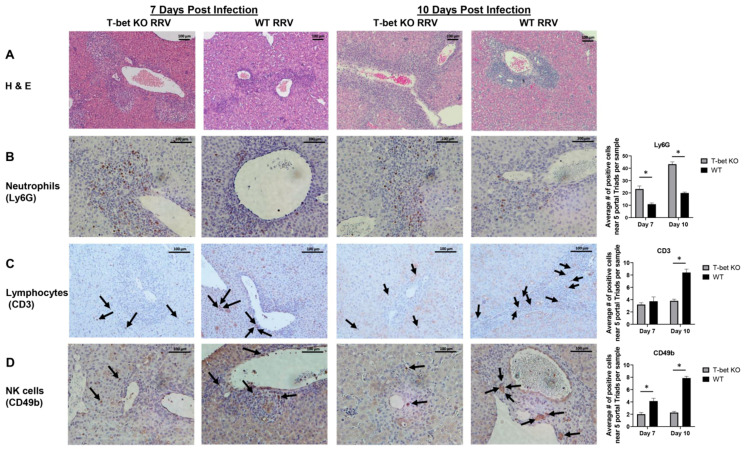
Histological and immunohistochemical staining of liver sections. Hematoxylin and eosin (H&E) staining of liver section both at 7 and 10 days post RRV infection revealed an increased inflammatory response in T-bet KO mice when compared to wild-type (WT) mice (**A**). Immunohistochemistry revealed a significantly greater neutrophil infiltration within the T-bet KO mice at both 7 and 10 days post infection (**B**), while WT mice had significantly higher levels of lymphocytes (**C**) and NK cells (**D**). Arrows indicating positive staining of CD3 or CD49b cells. Five portal triads were evaluated per sample with *n* = 3 per group. * *p* < 0.05. Abbreviation: Ly6G, lymphocyte antigen 6 complex locus G.

Histological examination of liver sections at 7 and 10 days after RRV showed infiltration of portal tracts with immune cells in both T-bet KO and WT mice demonstrated by immunohistochemistry (Figure 6A). Immunohistochemical staining for individual immune cell types revealed the cellular content in T-bet KO livers remained primarily as neutrophils at both time points, while RRV-injected WT livers contained primarily lymphocytes at 10 DPI and NK cells at both time points (Figure 6B–D).

### 3.7. Prevention of Extrahepatic Duct Obstruction in T-Bet KO Mice

**Figure 7:**
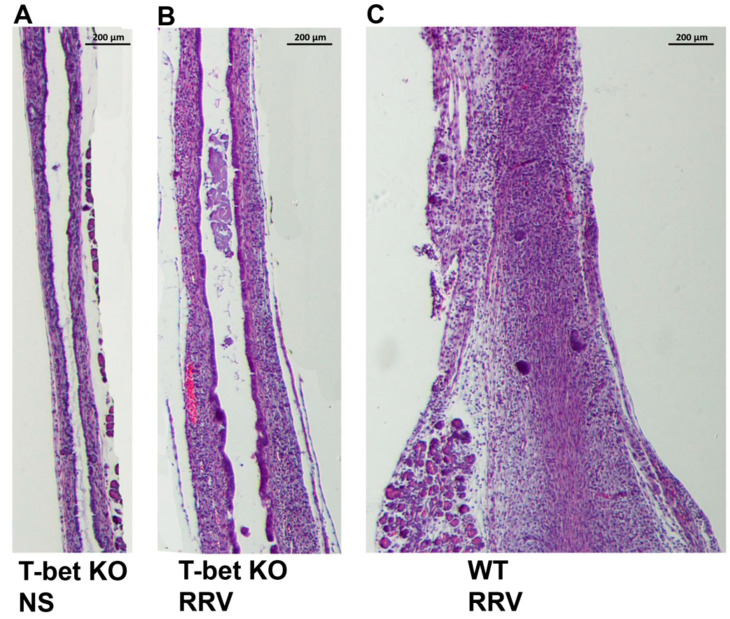
Histological analysis of bile ducts. Normal epithelium with unobstructed lumen in normal saline-injected (NS) T-bet KO controls (**A**). RRV infection leads to mild inflammation and a mostly patent bile duct in T-bet KO mice (**B**); in contrast, RRV infection leads to obstruction of the extrahepatic bile duct by inflammatory cells at day 12 in wild-type (WT) mice (**C**). Serial sections were stained with H&E.

In the murine model of BA, RRV infection in newborn pups leads to the complete obstruction of the extrahepatic bile ducts in WT mice by day 12. Interestingly, H&E-stained sections of extrahepatic bile ducts from T-bet KO mice showed mostly intact epithelial surface and a patent lumen despite mild subepithelial inflammation (Figure 7).

### 3.8. Suppression of Th1 Response Led to Impaired Viral Clearance in T-Bet KO Mice

**Figure 8:**
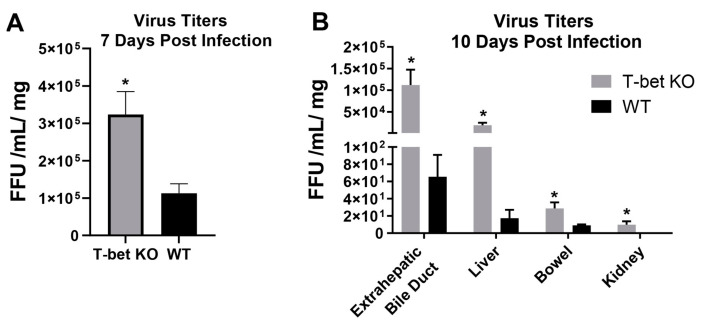
Quantification of RRV in tissue 7 and 10 days post infection. Virus titers analyzed from extrahepatic bile ducts harvested from day 7 revealed a significantly greater amount of RRV in the T-bet KO mice compared to the wild-type (WT) mice (**A**). At 10 days post RRV infection, T-bet KO mice had significantly higher titers in all organs investigated (**B**). * *p* < 0.05.

Previously it has been shown that the peak of rotavirus infection within the extrahepatic bile duct of newborn mice is at day 7 post infection [10]. In an effort to determine if the mortality witnessed in the T-bet KO mice was due to an increased viremia, we quantified the amount of virus in the extrahepatic bile duct at 7 and 10 days post infection (Figure 8A). At both the time points, the amount of RRV present in the extrahepatic bile ducts of T-bet KO mice was significantly greater than WT mice. Surprisingly, 10 days post infection, the T-bet KO mice still had a significant amount of virus presence in all organs investigated, suggesting an inability of the T-bet KO mice to control virus infection (Figure 8B).

## 4. Discussion

In this study, our findings demonstrated that ablation of T-bet protects against the obstructive component of biliary atresia in the murine model, suggesting that T-bet is required to regulate inflammation and plays a vital role in pathogenesis of BA.

Previously published data show that RRV infection causes a Th1 immune profile in the livers of infected mice [21,24]. In BA, CD8 T cells and NK cells play a critical role in initiation of the inflammatory immune response in the effector phase of the disease [14,15]. As the transcription factor T-bet is integral to the differentiation of Th1 cells, it is not surprising that T-bet deficiency altered development in the murine model of BA. We found that T-bet deficiency resulted in a significant reduction in hepatic production of IFN-γ, TNF-α, IL-6, and IL-10 along with an immune profile favoring a Th2 phenotype. This suggests that T-bet deficiency alters hepatic injury by a Th1/Th2 imbalance.

The transcription factors T-bet and signal transducer and activator of transcription (STAT)-1 regulate the differentiation of IFN-γ-producing Th1 cells [25]. A previous study showed that there is residual T-bet protein in the Th cells of STAT1 KO mice that may allow for the induction of IFN-γ leading to experimental autoimmune encephalomyelitis (EAE) [25]. Another report also demonstrated T-bet expression in CD4 T cells from *Toxoplasma gondii* infected STAT1 KO [26]. Therefore, residual T-bet might play a pathogenic role in STAT1 KO mice that are not protected from BA [27].

Chemokines are essential for the recruitment of immune cells into the injured liver in BA. To better understand the mechanistic impact of T-bet deficiency on leukocyte recruitment and liver injury, we analyzed chemokine expression within the liver. In the T-bet KO mice we observed a significant reduction in intrahepatic mRNA expression of the Th1 cell chemoattractants, CXCL2/MIP-2a, CXCL10/IP-10, and CCL5/RANTES. Conversely, an increase in intrahepatic mRNA expression of CCL17/TARC, which is a Th2 chemoattractant, was detected after T-bet deficiency, resulting in the increased expression of IL-4 and IL-13 in the liver. Not only did we see these differences in Th1 and Th2 chemokines, but we also observed similar differences in the expression of effector molecules in MNC, with a decreased expression of granzyme-A, perforin, and FasL, and increased levels of IL-4, IL-17, and IL-21. The total number of CD3, CD4, and CD8 T cells were also significantly lower in RRV-infected T-bet KO mice compared to RRV-infected WT mice. In addition to these differences in cell expression, liver inflammation of T-bet KO mice constituted a neutrophilic infiltration compared to lymphocytic in RRV-infected WT mice.

The onset of symptoms and mortality in T-bet KO mice was similar to that of WT mice; however, it is more important to note that greater than 50% of T-bet KO infected mice had a patent bile duct with mild inflammation compared with WT mice. This suggests that T-bet deficiency does alter the obstructive aspect of the murine model of BA. What remains to be determined is the basis for liver injury without an obstructed bile duct. We postulate that this may be because of the inability to clear the virus, leading to a higher virus load in T-bet KO mice.

## 5. Conclusions

The data in the current study demonstrate that genetically programmed biases in T cell subset differentiation and cytokine expression profiles influence pathogenesis of BA. The proinflammatory response of Th1 cells, which are suppressed in T-bet-deficient mice, alters the Th1 phenotype to a Th2 phenotype ultimately playing a protective role in experimental BA. Therefore, we conclude that downregulation of T-bet in the acute inflammatory response could be a protective strategy for reducing inflammatory cell infiltration and cytokine release, which may contribute to spontaneous self-remission of BA inflammation. More investigations should be performed to elucidate the mechanisms of T-bet involvement in regulation of BA pathogenesis.

## Data Availability

The data presented in this study are available on request from the corresponding author.
